# Langerhans Cell Histiocytosis of the Thyroid with Multiple Cervical Lymph Node Involvement Accompanying Metastatic Thyroid Papillary Carcinoma

**DOI:** 10.1155/2014/184237

**Published:** 2014-10-02

**Authors:** A. Bahar Ceyran, Serkan Şenol, Barış Bayraktar, Şeyma Özkanlı, Z. Leyla Cinel, Abdullah Aydın

**Affiliations:** ^1^Department of Pathology, Göztepe Research and Training Hospital, Istanbul Medeniyet University, Turkey; ^2^Department of General Surgery, Göztepe Research and Training Hospital, Istanbul Medeniyet University, Turkey

## Abstract

A 37-year-old male case was admitted with goiter. Ultrasonography of thyroid showed a 5 cm cystic nodule in the left lobe with a 1.5 cm solid component. Fine needle aspiration biopsy revealed atypia of undetermined significance or follicular lesion. The patient was operated on. The pathological diagnosis was reported as papillary thyroid carcinoma. The immunohistochemical examination showed multiple foci of Langerhans cell histiocytosis involving both lobes. The patient died due to cardiac arrest with respiratory causes in the early postoperative period. Langerhans cell histiocytosis is a rare primary condition which involves abnormal clonal proliferation of Langerhans cells in various tissues and organs. Thyroid involvement is infrequently seen. Although the etiology is unknown, genetic components may be linked to the disease. It is also associated with a family history of thyroid disease. Papillary thyroid carcinoma is the most common malignant epithelial tumor of the thyroid gland. Langerhans cell histiocytosis presenting with papillary thyroid carcinoma is rare. The privilege of our case is langerhans cell histiocytosis of the thyroid with multiple cervical lymph node involvement accompanying cervical lymph node metastatic thyroid papillary carcinoma.

## 1. Introduction

Langerhans cell histiocytosis (LCH) is a rare clonal proliferative disorder of the antigen presenting Langerhans cells with various tissue and organ involvements. The etiology remains to be elucidated with unknown pathogenesis [1–6]. In recent years, some have suggested an association between LCH and genetic components. The incidence of the disease increases with a family history with a higher incidence in monozygotic twins. It is also associated with a family history of thyroid disease. There is no evidence for viral etiology [4]. The incidence of LCH is 4–5.4/1.000.000 (mean 5) annually [1]. It is mostly seen in childhood and the male-to-female ratio is 3.7 : 1. Solitary or multiple lesions in an organ system may present or a generalized disease may develop with multiple organ involvement [3–6]. All organs may be affected by LCH [2]. Clinical manifestations of the disease may vary according to the involvement site [4, 6, 7]. The disease is graded according to the system involvement [1]. It may affect the skin, bones, lungs, hypothalamus, pituitary gland, lymph nodes, liver, spleen, and bone marrow [5, 6, 8].

Langerhans cell histiocytosis may present with several neoplasms in childhood and adulthood. Poor prognostic factors include the age of onset before 2 years, vital organ involvement including the liver, spleen, and bone marrow, and ≥3 involvement [4]. Thyroid involvement is infrequently seen. Review of the literature in English revealed 65 case reports of LCH presenting with thyroid involvement [1].

Papillary thyroid carcinoma (PTC) is the most common malignant epithelial tumor of thyroid gland. It is more common in women. Although it affects all age groups, the mean age at diagnosis is 40 years. It is associated with the external radiation treatment of the neck. Despite increased global incidence, the mortality has been decreasing. Prognosis is good in patients aged ≤45 years (more than 90%). One-fourth of patients have extrathyroidal involvement including the soft tissues of the neck. Cervical lymph node involvement is frequent in young patients in particular and may be the initial manifestation of the disease [3, 5].

Langerhans cell histiocytosis of thyroid presenting with metastatic PTC is extremely rare. There are few case reports in the literature [3, 9–14]. In this paper, we present a case of LCH of the thyroid with multiple cervical lymph node involvement presenting with cervical lymph node metastatic PTC.

## 2. Case Report

A 37-year-old man was admitted with goiter with a medical history of bullous lung disease and spontaneous pneumothorax. Ultrasonography of thyroid showed a 5 cm cystic nodule in the left lobe with a 1.5 cm solid component in the central of the nodule with peripheral calcification. An ultrasound-guided fine needle aspiration biopsy (FNAB) was performed. Cytological examination of the specimens revealed atypia of undetermined significance or follicular lesion (Hurthle cell-oncocytic type). Repeated biopsy was indicated, if clinically or radiologically required or surgical excision was recommended. A year later, the patient underwent left total thyroidectomy and intraoperative frozen-section biopsy of the left thyroidectomy revealed a classical variant thyroid carcinoma. Then, right total thyroidectomy and left modified neck dissection were concomitantly performed.

Grossly, 7 × 5 × 3 cm left lobe and 5.5 × 3 × 2 cm right lobe tissue of encapsulated thyroid were seen. A 4.5 cm nodule in a more semicystic nature with a 3 cm papillary site with smooth margins of the left lobe, a 0.8 cm cream-beige colored focus with irregular margins, and 0.2-0.3 cm cream-beige colored micronodular foci in the midline of the right lobe were detected. A total of 39 lymph nodes were found in the specimens taken during the left modified neck dissection.

**Figure 1:**
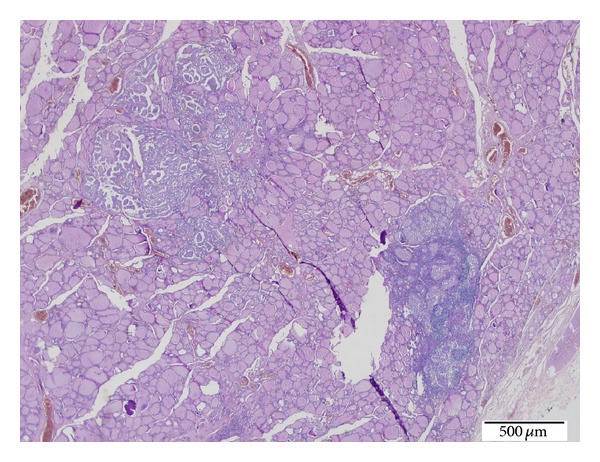
LCH foci presenting with PTC focus of the thyroid tissues (H.E. ×20).

**Figure 2:**
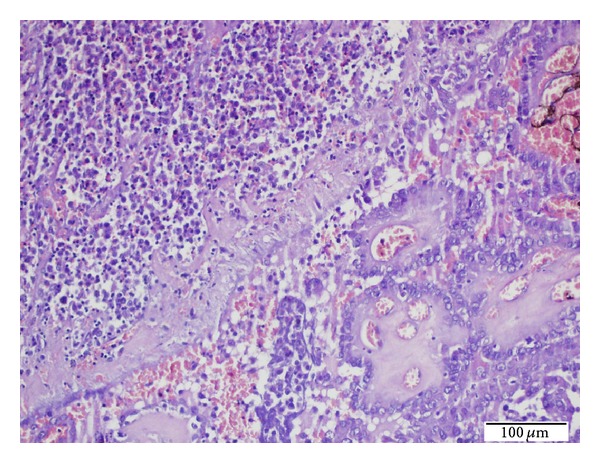
High power view of LCH foci (left upper corner) presenting with PTC focus (right lower corner) of the thyroid tissues (H.E. ×200).

Microscopic diagnosis revealed a 3 cm classical variant thyroid carcinoma in the left lobe nodule, a 0.3 cm papillary microcarcinoma in the cream-beige colored focus located in the right lobe, and multiple LCH foci associated with papillary carcinoma in both the lobes (Figures 1 and 2). There was no invasion in the surgical margins, thyroid capsule or surrounding tissues, and adipose connective tissues (pT1a). No lymphovascular invasion was observed, either. Chronic lymphocytic thyroiditis was detected. Four of the total 39 lymph nodes collected during the modified neck dissection had metastatic PTC with LCH foci involving all lymph nodes.

**Figure 3:**
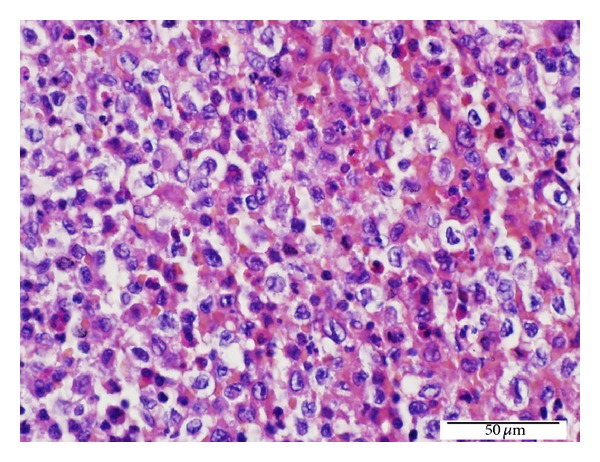
High power view of LCH foci in the thyroid tissues. Langerhans cells in granuloma-like groups and a number of eosinophil leukocytes (H.E. ×400).

**Figure 4:**
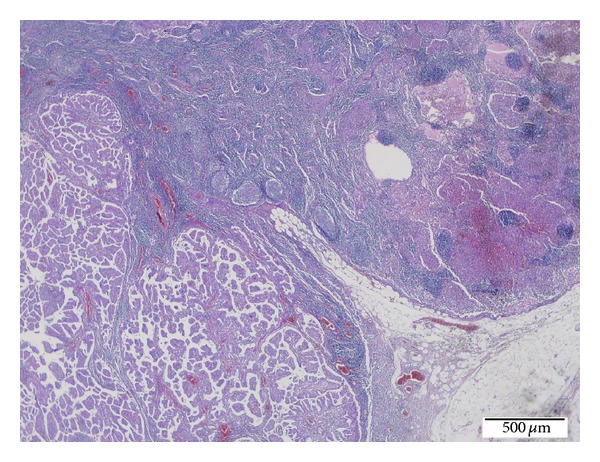
Metastatic PTC foci presenting with LCH within the same lymph node (H.E. ×40).

**Figure 5:**
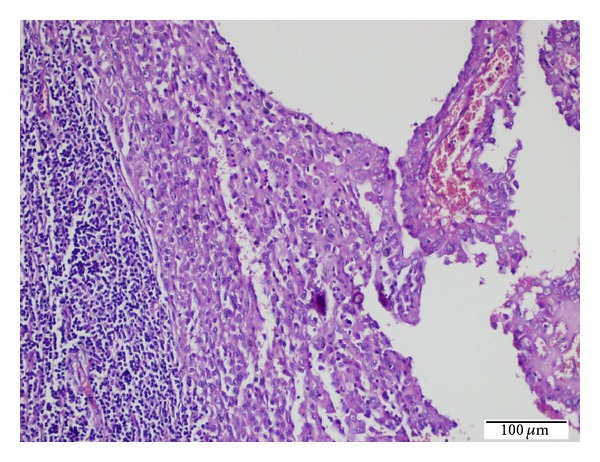
High power view of metastatic PTC foci (right side) presenting with LCH (middle) within the same lymph node (H.E. ×200).

**Figure 6:**
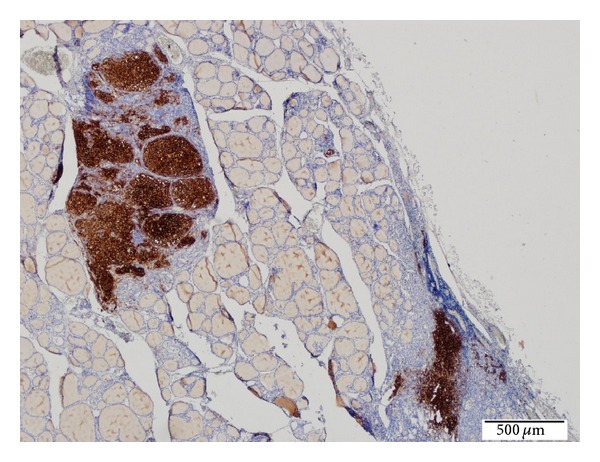
Positive CD1a-stained LCH foci in the thyroid tissues (Immunohistochemical technique, ×40).

**Figure 7:**
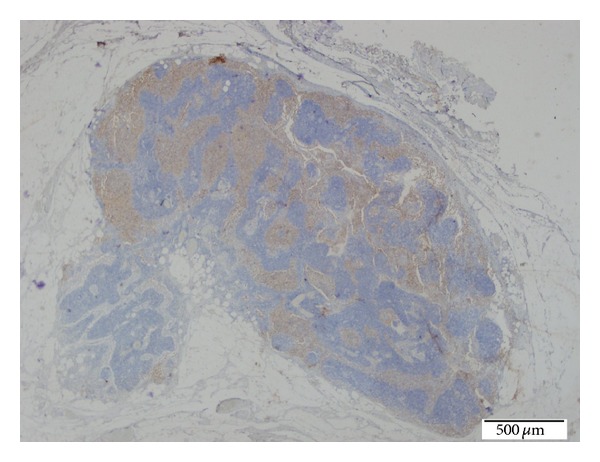
Positive CD1a-stained LCH foci in the lymph node. (Immunohistochemical technique, ×20).

Microscopic examination of LCH foci of both thyroid lobes showed papillary carcinoma foci presenting with diffuse chronic lymphocytic thyroiditis and granuloma-like groups. Granuloma-like groups consist of an abundant number of eosinophil leukocytes and Langerhans cells. Langerhans cells have abundant pale eosinophilic cytoplasm and vesicular nuclei with an indented “coffee bean” appearance (Figure 3). The lymph nodes demonstrated diffuse LCH foci adjacent to the metastatic sites in metastatic papillary carcinoma lymph nodes and sinusoidal invasion of LCH in nonmetastatic papillary carcinoma lymph nodes (Figures 4 and 5). The immunohistochemical analysis showed a positive staining with S-100 and CD 1a in the Langerhans cells in all involved sites (Figures 6 and 7). There was no intrathyroidal lymphatic invasion of PTC with D2-40 stain.

The patient was extubated postoperatively; however, he was reintubated due to respiratory distress in the intensive care unit. The patient remained unconscious with sedative effects. Plain X-ray showed no image consistent with pneumothorax. A bullous appearance was present in bilateral lungs with a diffuse parenchymal injury. The patient was administered sedative and prednisolone. The patient died due to cardiac arrest with respiratory causes in the early postoperative period. Autopsy could not be performed because his family did not allow it.

## 3. Discussion

Langerhans cell histiocytosis is rare with thyroid involvement. Although the etiology is unknown, genetic components may be linked with the disease [4]. Additionally, one study showed that 54% of the cases had BRAF V600E mutations [15].

Papillary thyroid carcinoma is the most common endocrine gland neoplasm with a high rate of BRAF V600E mutations [16, 17]. Several studies suggested that the BRAF V600E mutation might be associated with a poor prognosis of PTC along with a more aggressive infiltrative pattern [18–22], while some studies concluded that BRAF V600E mutations had no adverse effect on the disease prognosis [23–25].

LCH of the thyroid presenting with metastatic PTC cases in the literature is extremely rare [3, 9–14]. These case reports are only LCH of the thyroid presenting with cervical lymph node metastatic PTC. There is no LCH in the same metastatic lymph node in that case. However, our case is LCH of the thyroid with multiple cervical lymph node involvement presenting with same cervical lymph node metastatic PTC. To our best of knowledge, this is the first report in the literature.

In our case, multiple LCH foci presenting with PTC of the thyroid tissues were observed. Diffuse multiple LCH and invasive PTC foci were present in the cervical lymph nodes. The association of LCH with PTC, which is rare, can be attributed to the fact that the same mutation may play a role in the underlying pathogenesis of both diseases.

Previously, a number of Langerhans cells clustered around the malignant papillary cell groups, as evidenced by cytological examination of FNAB specimens of PTC, were reported. It was suggested that the presence of Langerhans cells might be a useful criterion for the diagnosis of malignancy [26]. The presence of polyclonal cells of LCH is typical with rare monoclonal cells and simultaneous presence in the same infiltrate was interpreted as the disease originating from polyclonal process or reactive infiltration, even progressing to monoclonal tumor infiltration [4].

In our case, we performed FNAB before the diagnosis of PTC, which revealed atypia of undetermined significance. Repeated examination of cytological specimens showed Langerhans cells as well as atypical cells. Taking the rare occurrence of LCH presenting with thyroid involvement into consideration, therefore, a thorough examination should be performed for the identification of Langerhans cells.

Pulmonary involvement is very common in older children or young adults in LCH and is usually a single organ phenomenon [4]. The lung can be involved in disseminated multivisceral LCH in young children [4, 27]. Most adult patients with pulmonary LCH (>90%) are smokers. Smoking can rapidly reinduce LCH in adults who had LCH in childhood [27]. It may also present with cervical lymph node involvement, resulting in a late addition, and spontaneous pneumothorax may develop before the diagnosis of LCH [28]. In this case, our patient had a known history of spontaneous pneumothorax. The patient died due to pulmonary complications with a poor prognosis in the postoperative period. The histopathological examination of thyroid tissues and lymph node specimens, which were collected postoperatively, was reported as LCH involvement. However, it was unlikely to establish whether the known pulmonary disease was associated with LCH. It is likely to have LCH lesions in the lungs before thyroid and lymph node involvement. Multisystemic involvement, pulmonary involvement in particular, is a high-risk condition with a poor prognosis [6]. The postoperative pulmonary complications with a poor prognosis can be attributed to this fact in our case.

## 4. Conclusion

LCH presenting with thyroid involvement is rare and can be diagnosed only histopathologically. It may be accompanied by certain types of thyroid diseases and PTC or metastatic PTC, in particular. LCH should be considered in the presence of Langerhans cell groups along with PTC, as assessed by both thyroidectomy specimens and cytological materials.

Once LCH presenting with thyroid involvement is detected, pulmonary involvement should be also investigated in patients with cervical lymph node involvement and pulmonary symptoms with a history of smoking. Novel and effective therapeutic management strategies should be applied to such patients with pulmonary involvement, considering rapid disease progression and poor prognosis.
